# Academy of Stomatology

**Published:** 1900-03

**Authors:** 


### ACADEMY OF STOMATOLOGY.

A REGULAR meeting of the Academy of Stomatology was held at the rooms of the Academy, 1731 Chestnut Street, Philadelphia, on the evening of November 28, 1899, the President, Dr. E. C. Kirk, in the chair. A lecture illustrated by lantern slides, upon the subject of "Comparative Odontography," was given by A. H. Thompson, D.D.S.

(For the report of Professor Thompson's lecture, see page 102.)

### DISCUSSION.

*President.*—Gentlemen, this subject is before you. It is a very interesting one. I hope all of you will take part in this discussion, and shall call upon Dr. Pierce to make some remarks, as he has interested himself in this matter for some time.

*Dr. C. N. Pierce.*—Mr. President: The illustrations have been remarkably interesting, giving us an admirable idea of what we can learn from the screen and the lantern. I think there is nothing that more fully illustrates its advantages for educational purposes. I shall not attempt to add anything to the statements that have been made, but there are some interesting freaks in evolutionary processes that may be mentioned. One was alluded to to-night. That there are deciduous incisors in the herbivora and not permanent incisors is a very remarkable modification of developmental processes. There is the sperm whale, with deciduous and permanent teeth. The great whale, or cachalot, has teeth in embryo which are partly developed and aborted, and as the animal reaches maturity we have those enormous baleen plates attached to the upper jaw for a distance of from ten to fifteen and twenty feet. These are peculiar freaks in nature which are interesting to study, and are hardly explained except by reason of modification of diet. The sperm whale, which lives on animal food, has only the cone-shaped teeth; while the balæna live on the smaller aquatic forms which become entangled in the fringes of the plates as the whales swim through the water with the mouth open. Then, another question is, whether the large cusp tooth we find in the herbivora especially is developed from spurs from the cone-shaped tooth, or whether it is the cementing together of a succession of these cone-shaped teeth that has given us the large herbivorous tooth.

If we take, for example, the elephant's tooth, we find a large tooth protruding from the ramus, and apparently being pushed forward. It is not all developed at once, but the anterior part is pushed forward, then new plates are added to the posterior surface until we get a full normal size. There is an interesting specimen in the Academy of Natural Sciences. Now, is that the manner in which our cone-shaped teeth of the higher mammals originally developed? Were they cones simply cemented together, or were they produced by spurs from the cone-shaped tooth, broadening out the tooth as it became necessary to have a masticating or grinding tooth? These are questions that are worthy of examination, and they offer an unlimited field of study for every young practitioner. To me it has been one of the most interesting studies, and although I have given but little time to it, yet it has added more pleasure to my dental practice than any other subject.

The remarks to-night and the illustrations have been to me intensely interesting, because they have so fully illustrated the changes that have taken place from the simple to the complex tooth, and I thank Dr. Thompson very much, indeed, for the opportunity he has given us of seeing these illustrations and the remarks he has made.

*Dr. A. P. Brubaker.*—Mr. President: I do not know that I have anything new to say on the subject, except to express my appreciation of the admirable lecture by Dr. Thompson. As the slides, however, were passed before us, a query came into my mind which appears germane to the subject, and which I will ask Dr. Thompson to answer for me. It is well known at the present time that the modern mammalian tooth is a modified descendant of the simple cone-shaped tooth of the reptiles. It is generally believed that the cusps on the molar teeth, at any rate, have been due to differentiation, owing to certain mechanical causes connected with the life of the animal.

Now, the query I have in mind is this: Why the incisor teeth do not present cones, and what have been the forces which have changed the shape of a cone-shaped tooth to that of a chisel-shaped tooth at one period? That is one query that does not seem to me to be in accordance with the ordinary causes which have developed the molar teeth from the simple cone.

Another query which presented itself to my mind was, Is the incisor tooth a degenerated tooth? For instance, in the herbivora, for example the cow, we find that the incisor teeth in the upper jaw have disappeared. Is this lack of development a case of degeneration?

*Dr. A. H. Thompson.*—I cannot say why it is so. I did not touch upon the matter of evolution of tooth-form because, as Kipling says, "that is another story." I have a series of slides on that subject, which is to me one of the most interesting branches of the study. Why the horse should have upper incisors and the cow should not is something that we do not understand, and yet both subsist upon the same sort of food. The history of the whole story of the evolution of tooth-forms is a very interesting thing. To begin at the beginning, there are two principal theories in regard to the evolution of tooth-form. The Germans have what they call the concrescence theory, in which they hold that the mammalian tooth is formed by the fusion of the single cones of the teeth as found in the reptiles. You know the mammals, as a rule, have fewer teeth than the reptiles, and the concrescence theory of the Germans is that the upper molars are formed by a number of reptilian teeth being fused together to form the wide, masticating molar tooth. The American theory as propounded by Allen, Cope, Osborne, Scott, and others of the American school, defines what is called the "differentiation theory;" that the cusp is the offspring of the cingule which grows on the side of the cone-shaped tooth. Taking the proto-cone, the cusp or cingule is developed on the side of the tooth and becomes in time an additional cusp, so that beginning with the three-coned tooth, which is the primitive type of the molar, we have developed from that the later forms of molars. American paleontologists hold that there is no mistake about it, for the birth of the cusp has been observed. Take, first, the primitive cone-shaped tooth, the proto-cone, we have on the side of this proto-cone developed first small false cingules or cusps, or cones, which are the beginnings of the cusps and represent the first step towards their evolution, so the American paleontologists say we are in at the birth of the cusps. These teeth continue to grow until we have the three cones in a row, then the triangular type of molar which is found in the carnivora, the premolars, in which there is a single-bladed tooth with these three cones. The molars of the seals show the primitive type of these three cones in a row. These are the proto-cone, the meta-cone, and the para-cone of the new nomenclature. I wish our nomenclature were of this simple kind. This tricondont form of tooth is the primitive type of molar. It has so happened that in the evolution of the molar there was a movement of these cones, so the proto-cone moved to the inner side, and we have the original triangular molar to which I called your attention in the premolar of the opossum. We have in the human molar the proto-cone, the mesio-lingual cusp or cone, and the mesio-buccal cusp is the para-cone and the disto-buccal is the meta-cone. That is the primitive triangular molar. Now, in the processes of evolution, a new wing arose on this triangular molar which became the hypo-cone, the fourth cone. The oblique ridge is the remains of the border of the primitive triangular molar. The lower molar is developed in the same way, and these molars pass each other in the triangular form. In some types this remained quite sufficient for the purposes of mastication, but in the process of evolution, when it became necessary that food should be more thoroughly masticated, there came the development of what is called the heel upon the upper molar, by which arose the fourth cone or cusp. This was thrown out and the cone raised on it, extending the masticating power of the tooth, by allowing it to strike in the valley of the lower molar, like a pestle in a mortar. That is the theory of differentiation or evolution of cusps as held by American paleontologists, but the Germans hold to the concrescence theory; and although they have done very beautiful things in the study of embryology, they do not defend this theory. The Americans contend that the cusps were developed by evolution from the cingules that were raised upon the sides of the proto-cone. It is a very beautiful study, but it is a long story, and I shall not go into details.

The incisors were probably produced by the flattening of the cone, to produce a cutting edge by simply spreading out, not by differentiation. In the carnivora, where the cutting function is usurped, the incisors are mere rudiments. The cutting is done with the long-bladed premolars, not by the incisors. In the cow and horse and other herbivorous animals, the incisors have greater development.

*Dr. Pierce.*—I would like to ask Dr. Thompson if he does not think the mechanical force is somewhat of a factor. We have in the lower jaw a third class lever, with the greatest force applied to the molars and the force as lessened upon the anterior teeth, so that the tooth of the anterior part of the jaw must be a cutting tooth or a cone-shaped tooth to be of any use at all.

*Dr. Thompson.*—According to Dr. John Ritter, the mechanical force is the element that has produced tooth-form. In the carnivora the jaw is much shorter and the muscles are moved forward, so that the force can be applied more directly. Great force is applied to the anterior part of the jaw, or the jaws are shortened, in order to make the cutting-teeth nearer where the force is applied.

*Dr. James Truman.*—During the lecture I could not help recalling my earlier experiences in teaching. We did not have the lantern and other means of illustrating this subject to our classes. Dr. Pierce, Dr. Darby, and others who had this work to do can appreciate the difference. To me the lecture was very interesting, but I cannot quite comprehend the evolution theories that are extant, or the changes explained by either the concrescence theory or by the building up of the cusps, and why all this should occur through the simple fact of change of diet. For instance, I cannot comprehend why the molar tooth of the herbivora and ruminants have the cement and enamel in such arrangement as to be always rough for the comminution of food. Neither can I understand the change from the cone-shaped tooth of the mammoth to those of the elephant, in which we find the teeth and cones are worn off. Neither can I understand why the teeth of rodents should be made continuous in growth and be worn to a chisel-shaped form. Evolutionary theories do not seem to me to explain anything. Once in discussion with Professor Cope, I asked whether the changes that we notice in the human subject in the supernumerary teeth, for instance, were reversions of type? This he would not or could not answer. We have there the cone-shaped tooth, and it is a typical tooth. There is no change, and I would like Professor Thompson to say if he can explain that condition of things. That cone-shaped tooth is always of the same character, of the same type. Is it a reversion to the earlier form? Does it belong practically to the human subject? And yet we find it all the way through. It seems to me there is something lacking yet in the theories that undertake to build up cusps by attachments, as Dr. Thompson has delineated on the blackboard. I cannot quite comprehend it. Perhaps this is my lack of intelligence. I suppose there never will be the better explanation of the whole topic that some of us would desire. I am an evolutionist to some extent, but I cannot exactly understand the evolutionary theories. I cannot comprehend that the dog should have a tubercular molar posterior to the sectorial tooth because he has changed his food.

*Dr. Thompson.*—I should like to be able to reply to Dr. Truman, but, as he says, we are still in the dark, and a great many things he would like to know probably we shall never know. We would like to have a great deal more information. Referring to the supernumerary teeth of man, I believe, as he suggests, that they are to a certain extent reversions; for instance, we know that man has but thirty-two teeth and the typical mammal has forty-four. Man has in the process of evolution lost twelve teeth. Now these teeth sometimes reappear. Sometimes we have an incisor that is quite typical, quite like the other lateral incisors. Sometimes we have cone-shaped teeth that are almost like canines, come and disappear. Sometimes we have bicuspids that are typical third bicuspids; sometimes we have a fourth molar that appears, in the higher races, appearing as pegged-shaped teeth, mostly on the outside of the jaws. I have reason to believe that these are to a large extent reversions, or sporadic reappearances of dental organs. As to the effect of food upon the development of the jaws and teeth, I think there is no mistake. What you might call food selection has dictated the forms of jaws and teeth, because they are especially adapted to foods, and it must be that the tooth is adapted to the material and not the material to the tooth. I think there is good reason to suppose that it has been developed by the kind of food the animal has used. I wish that I could throw more light on the subject, but, as Dr. Truman well says, "we are still in the dark."

*Dr. I. N. Broomell.*—It occurs to me that one factor favoring the question of concrescence may be explained in this way. When we take the matter of the development of the incisors into consideration, we find that they develop from three separate globes or plates along the cutting edge of the tooth. These, in the beginning, are cone-shaped calcified spots, and we have a union of these separate plates in the crown. We really have in the beginning some imperfect cone parts to this tooth. We can also consider that we have two cones in the bicuspid tooth which have become united by concrescence.

*Dr. S. H. Guilford.*—One of the first papers that Dr. Thompson wrote appeared perhaps twenty or thirty years ago in the *Dental Cosmos,* and was upon the subject of "The Suppression of the Wisdom-Tooth." I would like to know whether, as the years went by, he has found any further facts to confirm him in the belief that the wisdom-tooth should be finally suppressed or eliminated.

*Dr. Thompson.*—I think that there is every evidence that the wisdom-tooth, along with some other disagreeable organs in our anatomy, is being eliminated, and that it may be classed with the appendix vermiformis and other useless organs.

*Dr. Pierce.*—Lamarck said a good many years ago that there is a law of nature that had a tendency to produce that shape and structure which give the most efficient service. I think that is true, and explains the changes that are constantly taking place in the direction of modification of the teeth. There seems to be an effort on the part of nature to produce that structure and shape which will give us the most efficient service. (Replying to Dr. Kirk.) He does not explain how the law acts nor draw attention to the principle back of it.

*Dr. Thompson.*—I have replied to everything as far as possible as I went along and cannot say anything more. I think the third molar is in process of suppression, *i.e.*, in the process of evolution, and that the upper lateral incisor is following in the same path, because we often have the upper lateral incisor suppressed. It is often deficient in form, and is the most erratic tooth as far as known. I want to thank you very much for the courtesy of your attention, and I hope I have contributed to your entertainment and instruction, but I do not think I have done much of the latter.

*Dr. James Truman.*—I would like to make a motion. It is not customary to thank our members for giving us valuable papers, but I move a vote of thanks to Dr. Thompson for his valuable lecture. (Carried.)

